# Predicting Enzyme Turnover Numbers and Enabling Rational Enzyme Evolution

**DOI:** 10.1002/advs.77789

**Published:** 2026-09-27

**Authors:** Fengya Ge, Xiangyang Ma, Jiyan Li, Mengxiang Wang, Fuping Lu, Yihan Liu, Lin Wang

**Affiliations:** ^1^ College of Artificial Intelligence Tianjin University of Science and Technology Tianjin P. R. China; ^2^ Key Laboratory of Industrial Fermentation Microbiology, Ministry of Education, Tianjin Key Laboratory of Industrial Microbiology, The College of Biotechnology Tianjin University of Science and Technology Tianjin P. R. China

**Keywords:** $k_{\text{cat}}$ prediction, cross‐attention, enzyme engineering, enzyme kinetics, laccase

## Abstract

Enzyme turnover number ($k_{\text{cat}}$) is a central kinetic parameter for biocatalysis, but experimental determination is low‐throughput and existing computational methods inadequately model enzyme–reaction interplay. Here, we introduced MCKcat, a deep learning framework that integrates multi‐scale convolutional feature extraction and cross‐attention to enable deep, reciprocal fusion of enzyme sequence and reaction fingerprint representations for accurate $k_{\text{cat}}$ prediction. We constructed MCKcat‐DB, a large‐scale dataset comprising 33 396 enzyme‐reaction‐based $k_{\text{cat}}$ data points, covering both wild‐type enzymes and a large number of mutants. MCKcat demonstrated competitive performance across diverse prediction scenarios on two benchmarks. A two‐step strategy was developed to engineer *Bacillus aryabhattai* laccase with synergistically improved thermostability and catalytic activity. Rational design generated 217 candidate thermostable mutants, followed by MCKcat‐based screening that identified 20 hits. Validation showed 15 mutants (75% positive rate) exhibited simultaneous enhancements in both thermostability and $k_{\text{cat}}$. A user‐friendly web server was provided to facilitate broad adoption. MCKcat establishes a robust, generalizable strategy for data‐driven $k_{\text{cat}}$ prediction and artificial intelligence‐assisted enzyme engineering.

## Graphical abstract

MCKcat, a deep learning framework combining multi‐scale convolution and cross‐attention, accurately predicts enzyme turnover number (*k*_cat_) by fusing sequence and reaction representations. It couples with rational design in a two‐step strategy for *Bacillus aryabhattai* laccase engineering, achieving a 75% positive rate with synergistically enhanced thermostability and activity. A companion web server for MCKcat enables broad adoption.

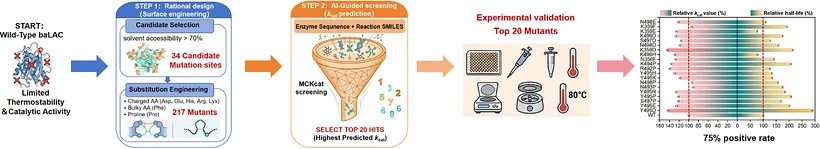

## 1 Introduction

Enzyme turnover number ($k_{\text{cat}}$) is a key kinetic parameter that quantifies the catalytic activity of an enzyme, representing the maximum number of substrate molecules converted to products per active site per unit time [1]. Accurate prediction of $k_{\text{cat}}$ values and rational ranking of the catalytic activity of enzyme mutants are critical for advancing enzyme engineering, synthetic biology, and industrial biocatalysis [2, 3, 4]. Traditional experimental determination of $k_{\text{cat}}$ is time‐consuming, labor‐intensive, and costly, especially for large‐scale screening of enzyme mutants and novel enzyme‐reaction combinations [5]. With the rapid development of machine learning and deep learning in computational biology, data‐driven models have emerged as powerful tools for predicting enzyme kinetic parameters, offering the potential to accelerate the discovery and engineering of high‐efficiency biocatalysts [6, 7].

Existing computational methods for $k_{\text{cat}}$ prediction, including DLKcat [8], UniKP [9], CataPro [10] and EITLEM [11], have made notable progress in integrating enzyme sequence and primary substrate information. It is worth noting that the $k_{\text{cat}}$ value is not merely determined by enzyme–primary substrate binding interactions but is governed by the entire catalytic reaction system, including co‐substrates, cofactors, products, conformational changes at the active site, and product formation pathways [12, 13, 14]. Using only substrate‐level representations results in the loss of essential reaction semantic information. By contrast, comprehensive reaction‐aware modeling enables more accurate characterization of the catalytic chemical transformation process, thereby boosting the model's generalization ability toward unseen enzymes. In light of this, TurNup [12] characterizes chemical reactions via numerical fingerprints that account for the complete set of substrates and products involved in a reaction, while CatPred [13] employs a concatenated SMILES string of all reactant molecules for $k_{\text{cat}}$ prediction. Despite achieving reasonable predictive accuracy for $k_{\text{cat}}$ values, these methods suffer from several inherent limitations: first, many methods simply extract enzyme and reaction/substrate features independently and perform naive concatenation, which is too shallow to capture fine‐grained catalytic interactions and often results in underfitting; second, others resort to computationally heavy unidirectional or multi‐head attention architectures, which introduce excessive model complexity and tend to overfit, especially given the limited size of high‐quality $k_{\text{cat}}$ data; third, many models exhibit poor predictive performance for non‐homologous enzymes and low‐similarity reactions—scenarios that are prevalent in practical enzyme engineering applications [15].

Among existing benchmark datasets for $k_{\text{cat}}$ prediction, the available reaction‐based $k_{\text{cat}}$ datasets comprise Kroll's dataset (TurNup‐DB) with 4271 data points [12] and CatPred‐DB with 23 197 data points [13]. Nevertheless, the overall size of these existing datasets remains relatively limited, and they lack coverage of enzyme mutants, which severely limits the generalization capacity of predictive models in directed evolution of enzymes.

**FIGURE 1:**
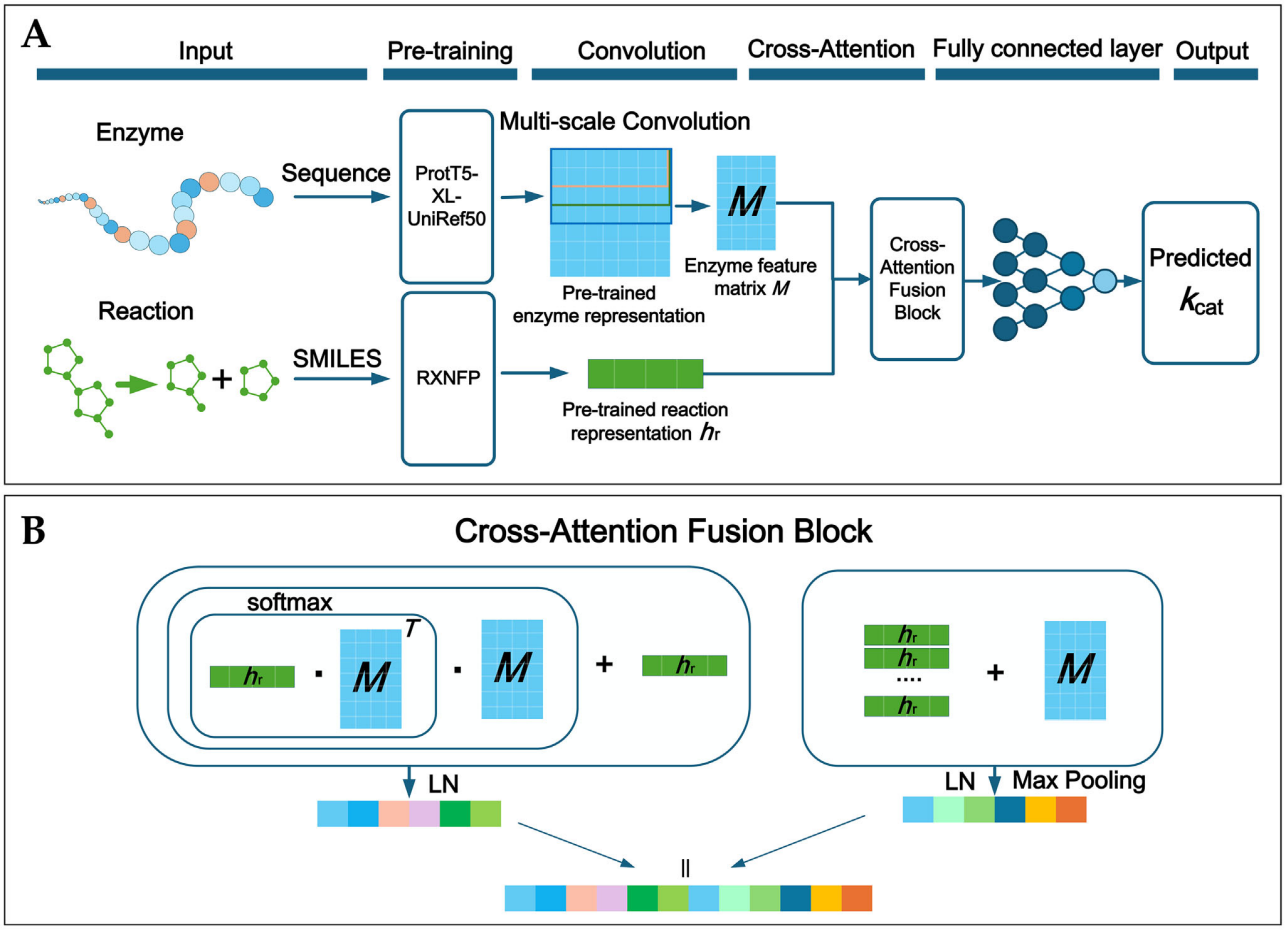
The framework of $k_{\text{cat}}$ prediction method MCKcat. (A) The framework comprises four core stages: input of enzyme sequences and reaction SMILES strings, feature extraction via pre‐trained representation learning and convolutional operations, cross‐attention fusion, and final prediction output. Specifically, the ProtT5‐XL‐UniRef50 model is used to generate pre‐trained representations for enzyme sequences, while the RXNFP model is employed to encode chemical reactions into corresponding pre‐trained embeddings. (B) Represents the core component of the model, which is responsible for the deep fusion of enzyme and reaction features. In the forward attention path, the pre‐trained reaction representation $h_{r}$ serves as the query, and the enzyme feature matrix M acts as both the key and value. After scaled dot‐product attention calculation, residual connection with the original reaction embedding, and layer normalization (LN), we obtain the optimized reaction embedding ${\hat{h}}_{r}$. In the reverse path, since the attention coefficient degenerates to 1 (uniform attention), the optimized enzyme feature matrix $\hat{M}$ is directly obtained by element‐wise addition of the original enzyme feature M and the reaction embedding $h_{r}$, followed by LN to stabilize feature distribution. Subsequently, max pooling is performed on $\hat{M}$ along the sequence dimension to obtain the vector representation of the enzyme. This vector representation is concatenated with ${\hat{h}}_{r}$ to form a complete enzyme‐reaction combined feature embedding.

To address these challenges, we proposed a novel multi‐scale cross‐attention fusion model, MCKcat, for $k_{\text{cat}}$ prediction (shown in Figure 1) and enzyme evolution. Distinct from methods relying on shallow feature concatenation or heavy attention mechanisms, MCKcat innovatively integrates multi‐scale convolutional feature extraction for enzyme sequences with a lightweight bidirectional cross‐attention mechanism. This design not only enables deep and mutual fusion of enzyme and reaction fingerprint features, accurately capturing the residue‐level interactions that govern catalytic activity, but also balances model complexity to avoid both underfitting and overfitting. Additionally, we constructed a large‐scale, high‐coverage enzyme–reaction‐based $k_{\text{cat}}$ dataset (MCKcat‐DB) with 33 396 data points comprising both wild‐type (WT) enzymes and numerous mutants, which substantially extended the data basis for model training and validation and further enhanced the model's generalization capacity in directed evolution of enzymes.

Comprehensive experiments on TurNup‐DB and our newly developed MCKcat‐DB confirmed that MCKcat outperformed state‐of‐the‐art methods across their respective key prediction scenarios. For unseen enzyme prediction on TurNup‐DB, MCKcat achieved a 13.86% higher $R^{2}$, 5.90% higher pearson correlation coefficient (PCC) and 10.14% lower MSE (Mean Square Error) than the second‐ranked method. It delivered a 4.33% higher $R^{2}$, 2.06% higher PCC and 6.57% lower MSE for unseen reactions. On the large‐scale MCKcat‐DB, it outperformed competitors by 3.73% in $R^{2}$ and 1.74% in PCC on the held‐out test set, with comparable performance on the out‐of‐distribution test set. MCKcat excelled at predicting non‐homologous enzymes and low‐similarity reactions on TurNup‐DB, reaching an average $R^{2}$ of 0.3744 for enzymes with 0% –20% sequence identity and 0.3313 for reactions with 0% –60% similarity. It also maintained competitive predictive performance for non‐homologous enzymes across all similarity ranges on MCKcat‐DB.

MCKcat also exhibited robust predictive power for enzyme mutant catalytic activity. On MCKcat‐DB, it achieved the highest average SCC (Spearman Correlation Coefficient) of 0.2146 for enzymes with ≥20 $k_{\text{cat}}$ values derived from mutants in the dataset, outperforming the next‐best method by 10.45%. Moreover, it demonstrated superior performance in identifying high‐fitness mutants across three deep mutational scanning (DMS) datasets (TEM‐1, BGL3 and HIS3), yielding SCC values of 0.4908, 0.2052, and 0.4748 respectively—an essential core capability for directed evolution of enzymes. We also developed a user‐friendly web server for MCKcat, which eliminates the requirement for specialized programming skills and thus facilitates its widespread use by the biochemistry and enzyme engineering communities.

Finally, we experimentally validated MCKcat's practical utility for rational enzyme engineering by engineering the laccase from *Bacillus aryabhattai* TCCC11368 (baLAC), an industrially promising biocatalyst limited by a typical stability‐activity trade‐off. We designed a two‐step rational engineering strategy: 217 candidate thermostable mutants were first rationally designed, then MCKcat was used for in silico screening to identify 20 top‐predicted hits with the predicted highest $k_{\text{cat}}$ values. Wet‐lab validation showed a 75% positive rate, with 15 mutants achieving synergistic improvements in both thermostability and $k_{\text{cat}}$ values. Y495D exhibited a 191.4% increase in thermostability and 42.8% higher $k_{\text{cat}}$ than the WT baLAC. This MCKcat‐guided in silico prescreening drastically narrows down the number of mutants requiring wet‐lab characterization, significantly reducing the cost and time of enzyme engineering and accelerating the development of high‐performance biocatalysts.

## 2 Results

### 2.1 Statistical Analysis of $k_{\text{cat}}$ Data

**FIGURE 2:**
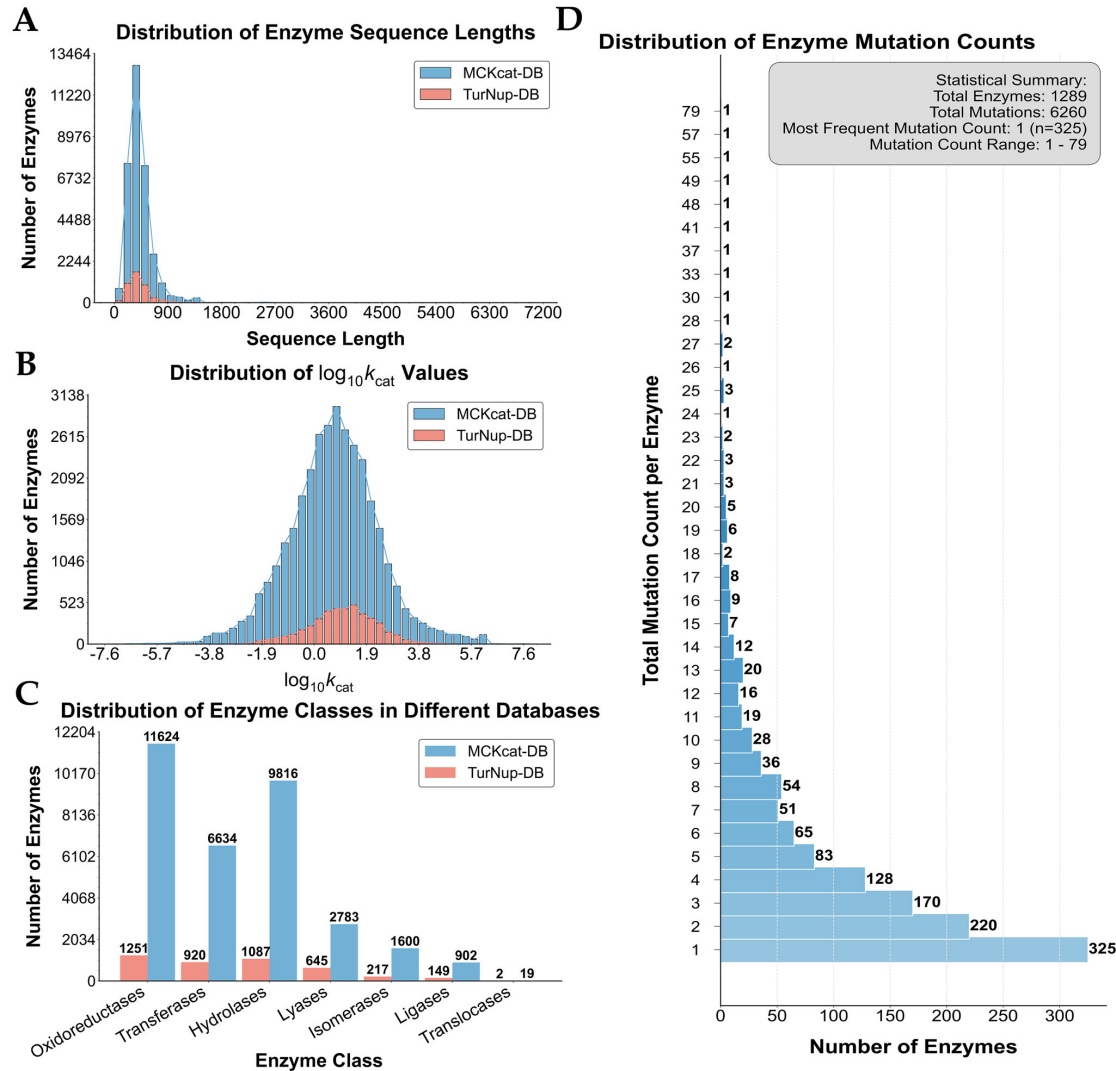
The comparison of statistical characteristics between TurNup‐DB and MCKcat‐DB. (A) Histogram of enzyme sequence length distribution for the two datasets. (B) Histogram of $\log_{10}k_{\text{cat}}$ value distribution for the two datasets. (C) Grouped bar chart of Enzyme Commission (EC) class distribution for the two datasets. (D) Horizontal bar chart of enzyme mutation count distribution in MCKcat‐DB, where the x‐axis represents the number of enzymes and the *y*‐axis represents the mutation count of enzymes.

TurNup‐DB and our newly developed MCKcat‐DB contain 4271 and 33 396 data points, respectively, with each data point corresponding to an enzyme, a catalytic reaction, and the associated $k_{\text{cat}}$ value. TurNup‐DB encompasses 2974 unique enzymes and 2800 distinct chemical reactions, while MCKcat‐DB includes 13 833 unique enzymes and 13 697 different chemical reactions. Here, unique enzymes are distinguished by full‐length sequences, covering WT enzymes and mutants. The enzyme sequence lengths in TurNup‐DB range from 12 to 2324 residues, and those in MCKcat‐DB from 7 to 7096 residues (Figure 2A). The $\log_{10}k_{\text{cat}}$ distributions of both TurNup‐DB and MCKcat‐DB exhibit unimodal characteristics, with mean values of 1.0223 and 0.7047, respectively (Figure 2B). MCKcat‐DB features a steeper peak and more concentrated distribution, whereas TurNup‐DB shows a broader and more dispersed pattern. Both datasets are dominated by oxidoreductases, transferases, hydrolases, and lyases, with fewer isomerases, ligases and translocases (Figure 2C). In addition, Figure 2D illustrates the distribution of mutation counts across 1289 enzymes in MCKcat‐DB. The dataset comprises a total of 6260 enzyme mutants, including 5270 single‐point mutants and 990 combinatorial multi‐site mutants.

### 2.2 Superior Performance for $k_{\text{cat}}$ Prediction

The TurNup‐DB comprises 4271 $k_{\text{cat}}$ values, encompassing 2974 WT enzymes and 2800 reactions. To validate the predictive performance of our MCKcat model, we first conducted experiments on the TurNup‐DB by implementing three data splitting strategies and comparing our model with four state‐of‐the‐art methods. The adopted train‐test data split strategies included: (1) Random Split: randomly dividing $k_{\text{cat}}$ values; (2) Unseen Enzymes: testing enzymes not seen during training; and (3) Unseen Reactions: testing reactions not seen during training. The baseline methods for comparison consist of TurNup [12], CatPred [13], CataPro [10], and EITLEM [11].

**FIGURE 3:**
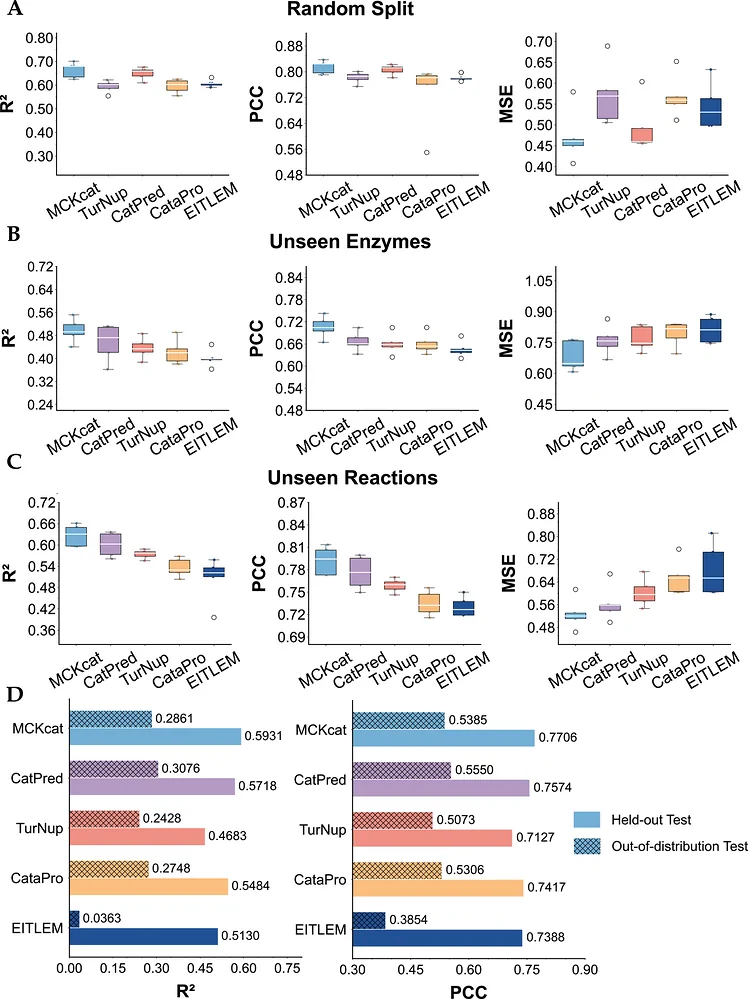
Comparison of Model Performance Across Different Tasks for TurNup‐DB and MCKcat‐DB. (A–C) are box plots for different tasks based on TurNup‐DB. The box represents the interquartile range of the data, the white horizontal line inside the box is the median, the ”whisker” represents the reasonable range of the data, and the white dots outside are outliers. (D) shows the $R^{2}$ and PCC of the five models on the held‐out test set and the out‐of‐distribution test set, respectively, based on MCKcat‐DB.

Specifically, in the case of the random split, we randomly partitioned the $k_{\text{cat}}$ values into an 80% training set and a 20% test set. This random splitting process was repeated five times, and the average performance over these repetitions was computed as the overall metric for comparison. This partitioning approach simulates scenarios where both enzyme and reaction information is available, serving to evaluate the model's predictive performance on commonly encountered enzymes and reactions. The test results showed that MCKcat achieved an average *R* 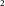 (Coefficient of Determination) of 0.6642±0.0292, PCC (Pearson Correlation Coefficient) of 0.8149±0.0179, and MSE (Mean Square Error) of 0.4728±0.0570, ranking as the top‐performing model among all tested ones (Figure 3A). CatPred showed an *R* 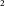 of 0.6496±0.0234, a PCC of 0.8063±0.0145, and an MSE of 0.4939±0.0565. TurNup (*R* 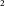 = 0.5943±0.0230, PCC = 0.7819±0.0160, MSE = 0.5723±0.0653), CataPro (*R* 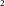 = 0.5958±0.0260, PCC = 0.7349±0.0930, MSE = 0.5681±0.0461) and EITLEM (*R* 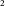 = 0.6079±0.0138, PCC = 0.7816±0.0091, MSE = 0.5449±0.0500) lagged behind.

In the unseen enzyme prediction scenario, the enzymes were partitioned into an 80% training set and a 20% test set, with this split repeated five times with different seeds. MCKcat obtained the best performance, achieving an average *R* 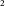 of 0.4969±0.0370, PCC of 0.7072±0.0262, and MSE of 0.6829±0.0653 across the five random splits (Figure 3B). In terms of *R* 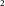 and PCC, MCKcat improved by 13.86% and 5.90%, respectively, while the MSE was reduced by 10.14%, compared with the second‐ranked method CatPred.

In the unseen reaction prediction scenario, MCKcat also demonstrated superior predictive performance with an average *R* 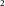 of 0.6271±0.0268, PCC of 0.7920±0.0167, and MSE of 0.5278±0.0487 across five random splits where reactions were partitioned into 80% training and 20% testing (Figure 3C). The test results indicated that MCKcat achieved a 4.33% improvement in *R* 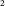, a 2.06% improvement in PCC and a 6.57% decrease in MSE compared to the second‐ranked method, CatPred.

To further quantify how each core module contributes to $k_{\text{cat}}$ prediction, we conducted systematic ablation studies targeting three key components: bidirectional cross‐attention, multi‐scale convolution modules, and convolution kernel combinations. We first validated the effectiveness of bidirectional cross‐attention by removing this module under unseen enzyme prediction and unseen reaction prediction scenarios. As shown in Figure S1, removing cross‐attention induced substantial performance degradation. For unseen enzyme prediction and unseen reaction prediction scenarios, the *R* 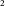 decreased by 16.18% and 39.34%, respectively. These results confirmed the effectiveness of the bidirectional cross‐attention module for $k_{\text{cat}}$ prediction.

We then investigated the functional impact of multi‐scale convolution and individual kernel sizes in the protein feature extraction branch through multiple controlled ablation variants. The modified model architectures were designed as follows: substituting the multi‐scale convolution block with a single linear projection layer, adopting a solitary convolution kernel (kernel size set to 2, 3 or 4), and implementing pairwise dual‐kernel combinations (2, 3), (2, 4), (3, 4). Across all evaluation tasks (shown in Figure S1), the full triple‐kernel multi‐scale convolution configuration achieved the optimal overall predictive performance, with the most prominent performance gain observed on the unseen enzyme scenario. This finding corroborated that multi‐scale convolution, together with the complementary representational power of diverse kernel sizes, was essential for capturing multi‐scale local structural motifs hidden in protein sequences.

### 2.3 Performance on a Large‐Scale Dataset

Since not only the primary substrate but also co‐substrates (i.e., secondary substrates, cofactors, etc.) contain information relevant to $k_{\text{cat}}$ prediction [13], the use of reaction information enables more accurate $k_{\text{cat}}$ prediction. CatPred has constructed a relatively large‐scale $k_{\text{cat}}$ dataset based on reactants; however, the dataset only includes WT enzymes, which limits its application in enzyme directed evolution. We integrated datasets from CatPred and CataPro to develop a larger $k_{\text{cat}}$ dataset (designated as MCKcat‐DB). This dataset contains 33 396 $k_{\text{cat}}$ values, among which 24 624 correspond to WT enzymes, 7424 to single‐point mutants and 1348 to multi‐site mutants. We validated the effectiveness of our model using MCKcat‐DB, with the specific validation protocol as follows: (1) The $k_{\text{cat}}$ values in MCKcat‐DB were randomly split into an 80% training set and a 20% test set. This test set, referred to as the held‐out test set, contains 6680 $k_{\text{cat}}$ values. (2) Furthermore, we filtered data from the held‐out test set under the criterion that each enzyme exhibited sequence identity no greater than 99% relative to any enzyme present in the training set. The resulting subset is referred to as the out‐of‐distribution (OOD) test set, which contains 639 $k_{\text{cat}}$ values. As shown in Figure 3D, our proposed method, MCKcat, achieved the best prediction performance on the held‐out test set, with its *R* 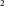 and PCC values exceeding those of the second‐ranked method, CatPred, by 3.73% and 1.74%, respectively. Furthermore, MCKcat attained comparable performance to baseline methods on the OOD test set, with an *R* 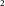 of 0.2861 and a PCC of 0.5385. Although slightly inferior to CatPred, it surpassed all other competing models.

### 2.4 Performance for Non‐Homologous Enzymes

**FIGURE 4:**
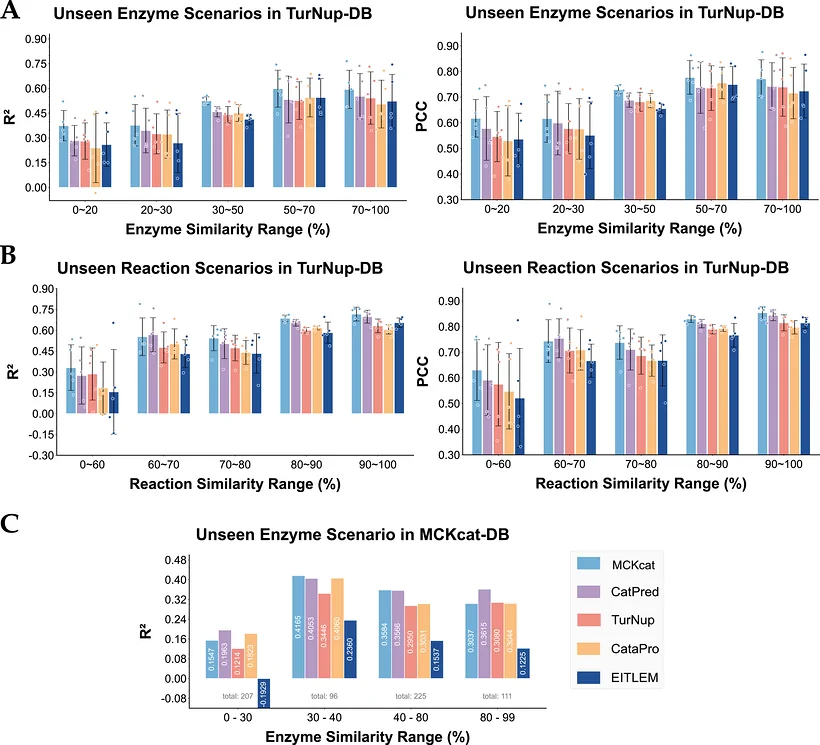
Performance Comparison in Low‐Similarity Scenarios based on TurNup‐DB and MCKcat‐DB. (A) and (B) are the *R* 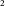 and PCC metrics under the unseen enzyme scenarios and unseen reaction scenarios, respectively, based on the TurNup‐DB. The scatter points represent the results of each of the five splits, the bars represent the indicator means of each model in the corresponding intervals, and the error bars represent the standard deviations. (C) shows the *R* 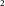 performance of five models across enzyme similarity intervals (0% –30%, 30% –40%, 40% –80%, 80% –100%) on the MCKcat‐DB.

To show the efficiency of the MCKcat model for non‐homologous enzymes, in the unseen enzyme prediction scenario for the TurNup‐DB, we divided the test set into five subsets based on the maximum sequence identity between the enzymes in the test set and the training set: 0% –20%, 20%–30%, 30%–50%, 50%–70%, and 70%–100%. Since we repeatedly split the TurNup‐DB five times using different random number seeds, we evaluated the prediction results of the same similarity interval across different splits. Figure 4A presents the comparison results of different methods across various enzyme similarity intervals in the TurNup‐DB. MCKcat maintained optimal performance across all similarity intervals and even exhibited significant advantages in the low‐similarity scenario (0% –20%). Specifically, it achieved average *R* 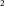 = 0.3744 and PCC = 0.6175, which were 32.30% and 6.87% higher than those of CatPred, the second‐ranking method, in this interval.

Furthermore, we used the large‐scale dataset MCKcat‐DB to demonstrate the $k_{\text{cat}}$ prediction capability of the MCKcat model for non‐homologous enzymes. We divided the enzymes in the OOD test set into four similarity ranges based on their maximum sequence identity to the enzymes in the training set, i.e., 0% –30%, 30%–40%, 40%–80%, and 80%–99%. Figure 4C illustrates the comparison results of different methods across various enzyme similarity intervals in the MCKcat‐DB. MCKcat achieved comparable predictive performance across different similarity ranges. Specifically, in the 30%–40% and 40%–80% similarity ranges, MCKcat surpassed all other competing methods.

Besides, we validated the predictive performance of MCKcat on low‐similarity reactions based on the TurNup‐DB. Specifically, in the unseen reaction prediction scenario, we divided the test set into five subsets based on the maximum reaction‐pair similarity between the test‐set and training‐set reactions, with similarity ranges of 0% –60%, 60%–70%, 70%–80%, 80%–90%, and 90%–100%. Similarly, we calculated various evaluation metrics for the prediction results of the same consistency range across different splits. Figure 4B shows that, as expected, model performance was better for reactions with higher similarity. However, even for low‐similarity reactions (0% –60%), the MCKcat model still exhibited superior predictive performance, achieving an average *R* 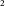 of 0.3313 and an average PCC of 0.6305. Notably, the average *R* 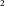 of EITLEM was 0.1567, indicating that its performance for reactions with low similarity was poor. This result indicates that the MCKcat model not only performs excellently in handling high‐similarity reactions but also demonstrates remarkable robustness for low‐similarity reactions, further highlighting its broad applicability in practical use cases.

### 2.5 MCKcat Enables to Rank Enzyme Mutants

An important application of $k_{\text{cat}}$ prediction lies in the directed evolution of enzymes. To validate the model's ability to rank enzyme mutants according to their $k_{\text{cat}}$ values, we filtered MCKcat‐DB to retain mutants whose corresponding parent enzyme had at least 20 $k_{\text{cat}}$ values derived from mutants (N≥20). Subsequently, we computed the sequence identity between the remaining enzymes and the previously retained ones. The remaining enzymes with a maximum sequence identity of ≤ 40% were then chosen as the training set to avoid overoptimistic performance estimation by including homologous enzymes. For each parent enzyme with N≥20, we calculated the Spearman correlation coefficient (SCC) between its predicted and experimental $k_{\text{cat}}$ values for its associated mutants. Notably, we used the SCC metric instead of the PCC metric, as the core goal of mutant screening is to correctly rank enzyme mutants by $k_{\text{cat}}$ rather than precisely predict absolute numerical values. Among parent enzymes with N≥20, the average SCC values achieved by MCKcat, CatPred, CataPro, EITLEM and TurNup were 0.2146, 0.0255, 0.1943, 0.1782, and 0.0280, respectively. Furthermore, regarding the fraction of parent enzymes with SCC greater than 0.4 within this group, the proportion for MCKcat reached 32.47%, which was higher than those for CataPro (23.38%), CatPred (7.79%), TurNup (10.39%), and EITLEM (28.57%).

Furthermore, we used Deep Mutational Scanning (DMS) data to expand the application scenarios of MCKcat for predicting the $k_{\text{cat}}$ of enzyme mutants. Here, we compiled DMS data related to the catalysis of three enzymes, namely TEM‐1 [16], BGL3 [17] and HIS3 [18]. Although the fitness values [19] obtained from their experiments are not equivalent to $k_{\text{cat}}$, they can be used to reflect the catalytic activity of enzymes. We used MCKcat‐DB as the training set and employed different prediction models to predict the $k_{\text{cat}}$ values for mutants within the three DMS datasets. We then compared these predicted values with the experimental fitness values to evaluate the models' ability to identify high‐fitness (HF) mutations.

**FIGURE 5:**
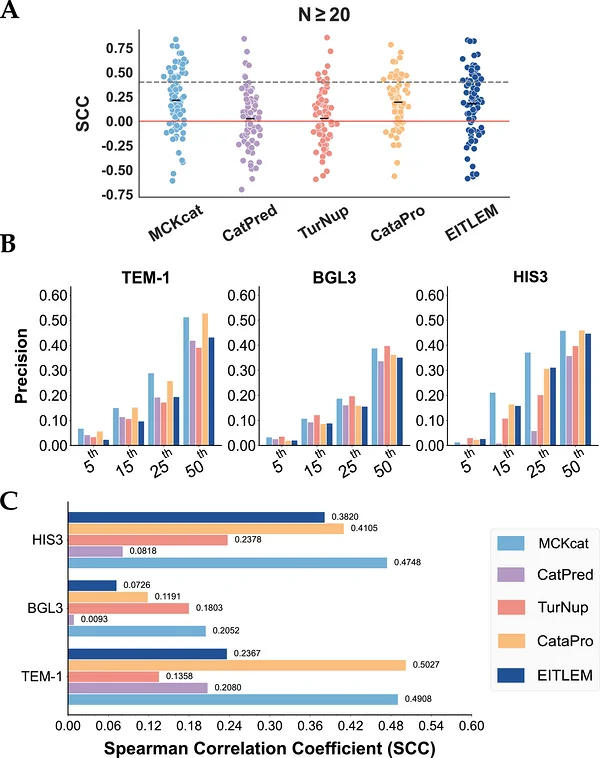
Performance comparison on enzyme mutants. (A) Shows the scatter plots of the Spearman's Correlation Coefficient (SCC) values in the scenario with N≥20. Circular scatter points represent the SCC values of each model across individual parent enzymes; black horizontal lines denote the mean SCC for each model. The red solid line indicates SCC=0, serving as the performance baseline, while the gray dashed line corresponds to SCC=0.4. (B) Shows the performance of different models in terms of prediction precision for identifying high‐fitness (HF) mutations based on predicted $k_{\text{cat}}$ values across the Deep Mutational Scanning (DMS) datasets of three enzymes. (C) Shows the comparison of SCC values of different models across the DMS datasets, which reflects the overall correlation between the predicted values and measured values of the models.

TEM‐1 is one of the earliest identified and most prevalent β‐lactamases in clinical isolates. It hydrolyzes the β‐lactam ring of ampicillin, a representative β‐lactam antibiotic, thereby conferring bacterial resistance to ampicillin. As such, it serves as a key target enzyme in studies of bacterial antibiotic resistance. A total of 5,198 TEM‐1 mutants were included in this analysis. We selected the top 5%, 15%, 25%, and 50% mutants ranked by MCKcat predictions, and the corresponding prediction accuracies for identifying the top 5%, 15%, 25%, and 50% fitness mutations of TEM‐1 were 6.80%, 15.07%, 28.93%, and 51.28%, respectively (Figure 5B). Relative to other models, MCKcat exhibited superior performance in identifying HF mutations of TEM‐1. Furthermore, the SCC between experimentally measured fitness values and $k_{\text{cat}}$ values predicted by MCKcat across all 5198 mutants reached 0.4908, showing performance comparable to CataPro (Figure 5C).

BGL3 (β‐glucosidase) is a key member of the glycoside hydrolase family. Its main function is to catalyze the hydrolysis of β‐glucosidic bonds, decomposing substrates, such as cellobiose and oligosaccharides into glucose monomers. It has important application value in fields such as cellulose degradation, food flavor improvement, and biofuel production. Since BGL3's catalytic efficiency directly affects the economy of relevant industrial processes, screening high‐fitness mutants of BGL3 is crucial for improving enzymatic performance. The dataset used in this study to evaluate the predictive performance of BGL3 mutants contained 26 653 data points, which covered comprehensive information on BGL3 mutants and their corresponding activity data–providing sufficient data support for verifying the reliability of prediction models. Under different threshold conditions, MCKcat exhibited a comparable ability to identify high‐fitness mutations of BGL3 compared to other models, with precision values of 3.34%, 10.77%, 18.80%, and 38.83% across percentile thresholds of 5^th^, 15^th^, 25^th^ and 50^th^, respectively (Figure 5B). Furthermore, across all 26 653 mutants, the SCC between experimentally measured fitness and MCKcat‐predicted $k_{\text{cat}}$ was 0.2052, a 13.81% improvement relative to the second‐best method TurNup (Figure 5C).

The main function of HIS3 is to catalyze a specific chemical reaction in the histidine biosynthesis pathway, specifically dehydrating imidazoleglycerol phosphate (IGP) to convert it into imidazolepyruvate phosphate (IAP) while releasing a water molecule. This reaction serves as the sixth step in the biological synthesis of histidine. The HIS3 DMS dataset contained 6,102 mutations. Compared with other models, MCKcat was better at identifying HF mutations, yielding precision values of 1.33%, 21.19%, 37.20%, and 45.88% across percentile thresholds of 5^th^, 15^th^, 25^th^ and 50^th^, respectively (Figure 5B). Furthermore, the SCC between the experimentally measured fitness values and the $k_{\text{cat}}$ values predicted by MCKcat across all 6102 mutants reached 0.4748, which corresponded to a 15.66% improvement over the second‐best method CataPro (Figure 5C).

### 2.6 MCKcat Assisted Enzyme Evolution

Enhancing the thermostability and $k_{\text{cat}}$ of known enzymes through protein engineering is a critical objective in biochemistry and synthetic biology. However, the interdependence of stability and $k_{\text{cat}}$ often presents a significant trade‐off, making it challenging to simultaneously optimize both properties during enzyme engineering [20, 21].

**FIGURE 6:**
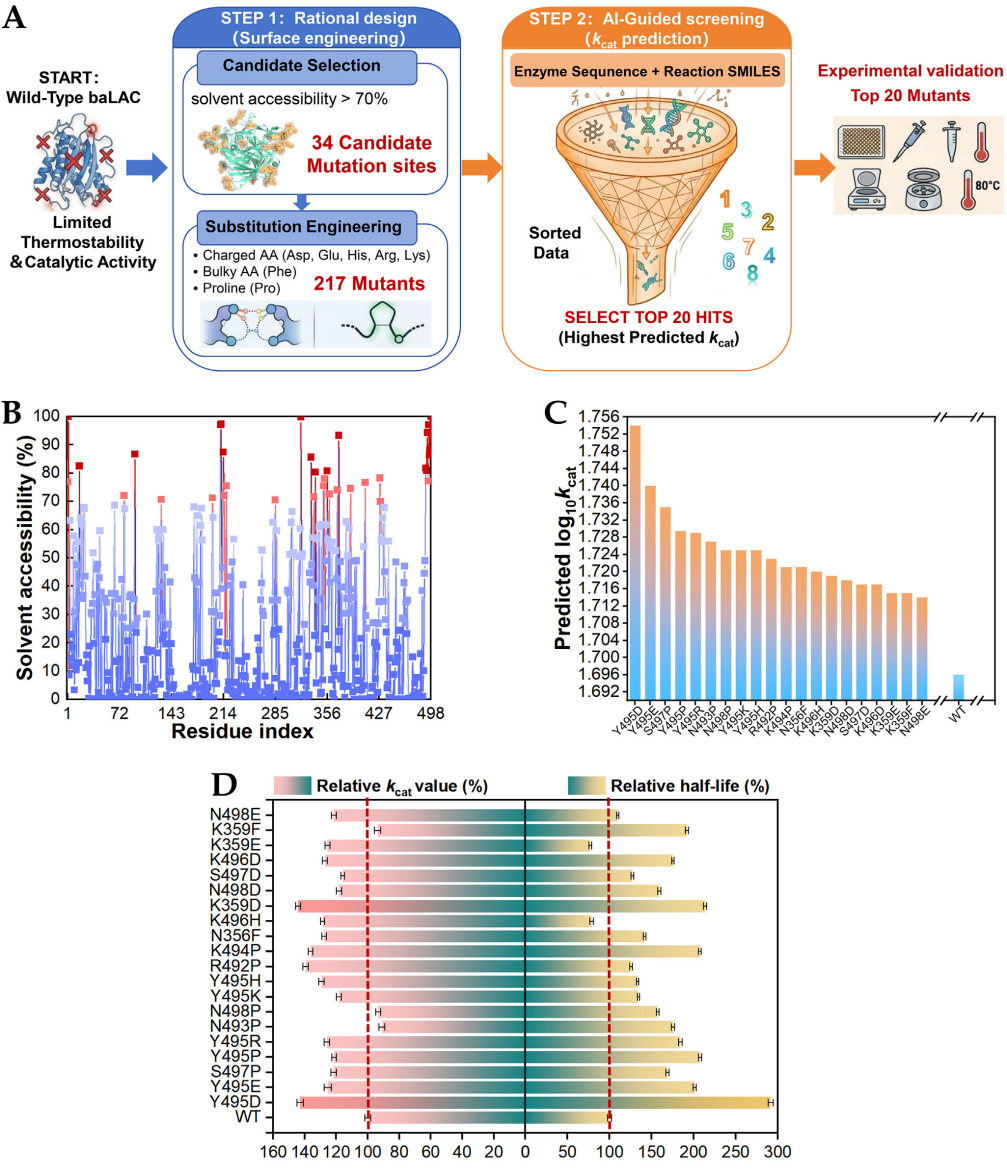
Prediction and virtual screening of hotspot residues for targeted mutagenesis to enhance thermostability and $k_{\text{cat}}$ values. (A) Workflow of the two‐step strategy for designing baLAC mutants. (B) The solvent accessibility profiles for all residues in WT baLAC were calculated with Dezyme (http://www.dezyme.com/). Residues labeled in red are designated as those with solvent accessibility over 70%, thereby serving as candidate mutation sites for protein engineering. **(C)** Predicted $\log_{10}k_{\text{cat}}$ values for the top 20 hits by using MCKcat. **(D)** Relative $k_{\text{cat}}$ values and half‐life of WT baLAC and its mutants.

Laccases have shown considerable promise for a range of industrial and environmental applications, including pulp biobleaching, degradation of persistent pollutants, development of electrochemical biosensors, and pretreatment of lignocellulosic materials [22]. However, the industrial application of natural laccases remains challenging, primarily due to their limited thermostability and catalytic activity. To address this issue, a two‐step strategy was implemented to design baLAC mutants with enhanced thermostability and catalytic activity (Figure 6A). Residues with solvent accessibility greater than 70% were considered surface‐exposed and presumed to have minimal influence on the catalytic activity (Figure S2). Accordingly, 34 residues (excluding the initial methionine) were selected as candidate mutation sites to minimize potential interference with catalytic activity (Figure 6B). Mutations at the 34 candidate sites were rationally designed based on three distinct stabilization mechanisms. Substitutions with charged residues (Asp, Glu, His, Arg, and Lys) were introduced at surface‐exposed positions to strengthen electrostatic interactions and promote salt‐bridge formation, both of which have been widely recognized as key contributors to protein thermostability [23]. In parallel, phenylalanine substitutions were incorporated at sites where the local structural context favored enhanced hydrophobic packing or aromatic stacking, thereby reinforcing inter‐residue contacts that stabilize the folded state [24]. Complementary to the surface electrostatic optimization and phenylalanine substitution strategies described above, proline substitutions, a well‐established thermostabilization strategy that reduces the backbone conformational entropy of the unfolded state and rigidifies locally dynamic segments, were specifically introduced [25]. Therefore, the above seven amino acids (Asp, Glu, His, Arg, Lys, Phe, and Pro) were considered to be thermostability enhancing types, and 21 out of the 34 candidate mutation sites fall into this category. After integrating all design strategies, a total of 217 single mutants (34 sites × 7 – 21 substitutions, excluding the 21 sites that already possessed these thermostability‐enhancing amino acids in the WT baLAC) were selected. The MCKcat tool was then employed to predict the $k_{\text{cat}}$ value for each mutant. From these predictions, the 20 mutants with the highest predicted $k_{\text{cat}}$ values were chosen for experimental validation (Figure 6C).

Protein expression experiments confirmed that all 20 mutants were expressed in soluble form (Figure S3). The mutants were subsequently purified (Figure S5) for assessing their $k_{\text{cat}}$ values and thermostability. As exhibited in Figure 6D, 85% of the designed mutants (17 out of 20) demonstrated higher $k_{\text{cat}}$ values than the WT baLAC. Among these, the K359D exhibited the greatest enhancement, with a $k_{\text{cat}}$ value 44.2% higher than that of the WT baLAC. These results verified that MCKcat provided an effective and rapid approach for predicting the $k_{\text{cat}}$ values of enzymes toward specific substrates. Next, the thermostability of all mutants was further assessed by measuring their half‐life ($t_{1/2}$) at 80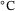. It was observed that 75% of the mutants (15 out of 20) demonstrated synergistic improvements in both thermostability and $k_{\text{cat}}$ in comparison to the WT baLAC. Among them, the Y495D exhibited excellent properties, demonstrating a 191.4% elevation in thermostability and a 42.8% elevation in $k_{\text{cat}}$ value compared to the WT baLAC. Collectively, these results validated that the two‐step strategy integrating MCKcat with rational design offered a pragmatic and scalable framework for AI‐guided protein engineering that substantially reduced experimental costs.

In addition, the 10 mutants with the lowest predicted $k_{\text{cat}}$ values were subjected to experimental validation (Figure S7A). Protein expression experiments confirmed that all 10 mutants were expressed in soluble form (Figure S4). The mutants were subsequently purified (Figure S6) for assessing their $k_{\text{cat}}$ values and thermostability. As shown in Figure S7B, 8 of the 10 lowest‐ranked mutants (80%) showed reduced $k_{\text{cat}}$ values compared with the WT baLAC, corroborating the capability of MCKcat to effectively identify $k_{\text{cat}}$ reducing mutants. Such bidirectional validation spanning both top‐ and bottom‐ranked predictions further confirmed the predictive accuracy and practical applicability of MCKcat in AI‐guided enzyme engineering. Meanwhile, 80% of the mutants (8 out of 10) displayed improved thermostability accompanied by reduced $k_{\text{cat}}$ values, further demonstrating the effectiveness of the two‐step strategy integrating MCKcat and rational design.

### 2.7 Web Server Facilitates Easy Use of MCKcat

We have deployed a web server that enables users to leverage the MCKcat model without requiring programming skills or specialized software installations (Figure S8). The server can be accessed at [http://117.72.109.140:5000]. This web‐based platform requires input of an enzyme's amino acid sequence, along with characterization data for all substrates and products. Substrate/product characterization can be provided using either SMILES strings or InChI strings. For batch processing capabilities, users need to upload a CSV file containing multiple reactions to execute calculations in bulk.

## 3 Discussion

The accurate prediction of enzyme $k_{\text{cat}}$ values and the rational ranking of mutant catalytic activities are pivotal for unlocking the potential of biocatalysis in industrial and biomedical applications. In this study, we developed MCKcat, a multi‐scale bidirectional cross‐attention network, and a large‐scale $k_{\text{cat}}$ dataset (MCKcat‐DB) to address the key limitations of existing computational methods. Our comprehensive experimental results demonstrated that MCKcat achieved state‐of‐the‐art performance in $k_{\text{cat}}$ prediction across diverse scenarios, and its unique bidirectional cross‐attention mechanism enabled deep modeling of enzyme‐reaction interactions–an advancement that overcame the feature fusion bottlenecks of previous models. Beyond predictive performance, MCKcat also excelled in ranking enzyme mutant activity and guiding rational protein engineering, as validated by both deep mutational scanning (DMS) data analysis and wet‐lab experiments on baLAC. These findings highlighted MCKcat's dual value as a computational prediction tool and a practical guide for experimental enzyme engineering, bridging the gap between in silico modeling and wet‐lab experimentation.

A core insight from this work was the critical importance of bidirectional feature fusion between enzymes and reactions for accurate $k_{\text{cat}}$ prediction. Unlike baseline methods that relied either on cumbersome unidirectional/multi‐head attention architectures or on naive feature concatenation (e.g., EITLEM, CatPred), MCKcat leveraged a lightweight bidirectional cross‐attention mechanism to enable the mutual refinement of enzyme and reaction features. Enzyme residue features were polished with insights from reaction fingerprint information, and reaction features were optimized by learning contextual details of enzyme sequences. This mutual learning process effectively captured the residue‐level interactions between enzyme active sites and reaction substrates/products–the fundamental molecular basis of catalytic activity–thus explaining MCKcat's superior performance for non‐homologous enzymes and low‐similarity reactions. Additionally, the integration of multi‐scale convolutional feature extraction for enzyme sequences further enhanced MCKcat's ability to capture local structural features of enzymes (e.g., active site motifs, conserved residues), complementing the global feature learning of the pre‐trained ProtT5‐XL‐UniRef50 model and improving the model's sensitivity to key catalytic residues.

The construction of MCKcat‐DB was another key contribution of this study, addressing the data scarcity and mutant underrepresentation issues of existing $k_{\text{cat}}$ datasets (e.g., TurNup‐DB and CatPred‐DB). MCKcat‐DB provided comprehensive coverage of major enzyme classes (oxidoreductases, transferases, hydrolases, lyases) and a wide range of sequence lengths and mutation counts. The large scale and high diversity of MCKcat‐DB not only improved the generalization ability of MCKcat but also provided a valuable public resource for the research community to develop and validate new $k_{\text{cat}}$ prediction models. Notably, MCKcat‐DB included a large number of enzyme mutant data, which was essential for training models to rank $k_{\text{cat}}$ values of mutants–a capability that was largely missing in existing models trained only on WT enzymes.

MCKcat's performance in enzyme mutant ranking and directed evolution further validated its practical utility. On DMS datasets of TEM‐1, BGL3, and HIS3, MCKcat outperformed baseline methods in identifying high‐fitness (HF) mutants, with high precision for top‐ranked mutants. For enzyme groups with sufficient mutant‐derived $k_{\text{cat}}$ measurements (N≥20), MCKcat achieved the highest average SCC and the largest proportion of enzymes with SCC >0.4, indicating its capacity to accurately rank $k_{\text{cat}}$ values of mutants. This capability is critical for directed evolution workflows, in which researchers needed to prioritize a small number of promising mutants for experimental validation.

The traditional method of screening mutants with high $k_{\text{cat}}$ from large mutant libraries through labor‐intensive and time‐consuming experiments hampers the development of enzymology and synthetic biology applications. The application of MCKcat to directed evolution of baLAC demonstrated its potential to revolutionize synthetic biology and biochemistry research. This study demonstrated that MCKcat could efficiently identify mutants with high $k_{\text{cat}}$ from 217 thermostable baLAC candidates. In baLAC directed evolution, it reduced the number of screened clones from 217 to merely 20, shortened the experimental cycle from several months to a few weeks, and directly cut the time and economic costs of industrial enzyme engineering, thus greatly accelerating the optimization of baLAC's $k_{\text{cat}}$. Among all mutants, Y495D was the most remarkable mutant, which achieved synergistic improvements in thermostability and $k_{\text{cat}}$ value, with a 191.4% enhancement in thermostability and a 42.8% increase in $k_{\text{cat}}$ compared with the WT baLAC. Moreover, the results showed that 17 of the 20 selected mutants exhibited higher $k_{\text{cat}}$ values than the WT baLAC, with a positive rate of 85%. By accelerating the enzyme engineering process, MCKcat is expected to serve as a powerful tool for advancing biocatalysis, metabolic engineering, and other enzyme‐dependent catalytic applications.

The development of a user‐friendly web server for MCKcat further democratizes access to this powerful tool, eliminating the need for specialized programming or machine learning expertise. By accepting enzyme amino acid sequences and reaction SMILES/InChI strings (with batch processing support via CSV files), the web server enables biochemists, enzyme engineers, and synthetic biologists to easily predict $k_{\text{cat}}$ values without any computational barriers.

Despite its strong performance in predicting $k_{\text{cat}}$ and ranking enzyme mutants, MCKcat exhibits several limitations that point to clear avenues for future improvement. First, data quantity and standardization remain limiting factors. Although MCKcat‐DB substantially expands available $k_{\text{cat}}$ data, coverage is still uneven across rare enzyme classes (e.g., isomerases, ligases and translocases). Experimental conditions such as pH, temperature, and ion strength are not uniformly annotated, and data noise persists across sources. Future work could integrate multi‐source databases, unify experimental metadata curation, and apply homology‐ and similarity‐based data augmentation to strengthen representation for under‐sampled enzyme–reaction pairs.

Second, given the input‐length constraint of the ProtT5 model, all protein sequences were uniformly truncated to the first 1000 amino‐acid residues during preprocessing. To validate this truncation strategy, we calculated the fraction of ultra‐long sequences (>1000 residues) in the TurNup‐DB and MCKcat‐DB datasets. Ultra‐long sequences account for only a negligible fraction: 1.24% (53 of 4271 data points) in TurNup‐DB and 2.54% (849 of 33 396 data points) in MCKcat‐DB. Truncation of ultra‐long proteins may discard C‐terminal structural segments and functional domains, potentially impairing feature extraction and prediction performance. Nevertheless, their very low abundance suggests that the overall adverse impact on our results is minimal and would not fundamentally undermine model reliability or generalization. This truncation scheme follows the preprocessing pipelines adopted by UniKP (ProtT5‐XL‐UniRef50) and TurNup (ESM‐1b). Consistent preprocessing removes confounding variables arising from divergent sequence‐handling protocols, guaranteeing fair and credible benchmark comparisons. Still, information loss for ultra‐long sequences cannot be fully disregarded. For future model improvements, we plan to explore dual‐branch embedding of N‐terminal and C‐terminal fragments as well as sliding‐window‐based full‐sequence representation approaches. The former reduces the risk of missing C‐terminal catalytic domains from naive N‐terminal truncation, whereas the latter captures information across full‐length ultra‐long proteins at the expense of higher computational overhead.

Third, model architecture is restricted to sequence‐level inputs. MCKcat does not incorporate enzyme 3D structures (e.g., from AlphaFold2) or reaction transition‐state information, which govern active‐site geometry, residue orientation, and substrate binding. Future designs could fuse sequence, evolutionary conservation, and predicted structural features with spatial cross‐attention to enable sequence–structure–reaction multi‐modal interaction modeling. Integrating molecular dynamics or docking data [26] will further capture dynamic conformational changes, moving beyond static representation to better reflect catalytic reality.

Fourth, robustness to distributional shifts can be strengthened. The model relies on pre‐trained protein language models and is sensitive to outliers and low‐homology cases. Domain‐adaptive fine‐tuning on enzyme kinetics data [27], combined with robust training pipelines and outlier filtering, could improve stability and prediction accuracy for distantly related enzymes and uncommon chemistries.

Finally, predictive scope is limited to single‐parameter $k_{\text{cat}}$ estimation. Practical enzyme engineering requires simultaneous inference of Michaelis constant ($K_{m}$) [28], catalytic efficiency ($k_{\text{cat}}/K_{m}$) [10], thermostability (Tm) [29], and solubility [3]. A unified multi‐task framework or platform that jointly predicts key kinetic and stability parameters will support comprehensive catalytic profiling.

## 4 Materials and Methods

### 4.1 Data Sets

The first dataset was sourced from TurNup [12]. TurNup integrated the turnover number ($k_{\text{cat}}$) values of enzyme‐reaction combinations from the Sabio‐RK [30], UniProt [31] and BRENDA [32] databases, and finally obtained 4271 data points. These data cover 2974 unique enzymes and 2800 unique reactions. Enzymes are characterized by their amino acid sequences, while the molecules in the reactions are represented by InChI strings. To meet the input requirements of our model, we utilized the RDKit library [33] to convert the InChI strings of each molecule into the SMILES format. Next, we connected the SMILES of chemical reactants and products with “>>”, which represents the generation relationship, and used “·” to separate components in reactants or products. In this way, we obtained the SMILES sequence of the entire reaction. In addition, to make the target variable approximate a Gaussian distribution and facilitate model training and optimization, we performed a logarithmic transformation of base‐10 on the $k_{\text{cat}}$ values.

Due to the small number of data points in TurNup, which includes only WT enzymes, the application capability of the model for enzyme‐directed evolution was limited. We integrated data from CataPro and CatPred to generate a new and larger scale $k_{\text{cat}}$ dataset. Specifically, the following three procedures were adopted: (1) CatPred offered 23 197 WT data points, each encompassing combined information on enzyme, reaction, and $k_{\text{cat}}$ value. For identical enzyme–reaction pairs with multiple source‐derived $k_{\text{cat}}$ values, CatPred designated the maximum $k_{\text{cat}}$ as the representative value of each pair; (2) From CataPro, we obtained 16 828 WT data points and 10 830 mutant data points, each containing combined information on enzyme, substrate, and $k_{\text{cat}}$ value. Similarly, for identical enzyme–substrate pairs with multiple source‐dependent $k_{\text{cat}}$ measurements, CataPro adopted the maximum $k_{\text{cat}}$ value as the representative value for that enzyme–substrate pair. For the substrate data in CataPro, we retrieved the corresponding reaction information from the Sabio‐RK and BRENDA databases. For reactions involving multiple substrates, the maximum $k_{\text{cat}}$ across all individual substrates was defined as the representative value of the enzyme–reaction pair originating from CataPro; (3) To integrate $k_{\text{cat}}$ data points collected from CatPred and CataPro, we standardized all reaction SMILES strings with RDKit. For identical enzyme–reaction pairs identified after SMILES normalization, the largest available $k_{\text{cat}}$ value was retained as the consolidated kinetic constant for the final MCKcat‐DB dataset. This process produced a comprehensive dataset containing 33 396 $k_{\text{cat}}$ values, covering 13 697 reactions, 7573 WT enzymes, and 6260 mutant enzymes.

### 4.2 Appropriate Representations for Enzymes and Reactions

#### 4.2.1 Enzyme Representations

By leveraging pre‐trained protein language models, such as ProtTrans, protein sequences can be transformed into high‐dimensional embeddings to accurately capture crucial biological signals. Among them, the ProtT5‐XL‐UniRef50 model [34, 35] is built on the Transformer architecture and can conduct unsupervised pre‐training on large‐scale protein sequence datasets, thereby generating embedding vectors rich in a large number of sequence features. Here, we used ProtT5‐XL‐UniRef50 to generate enzyme representations. Since the maximum number of amino acid residues that ProtT5‐XL‐UniRef50 can handle is 1000, for too long enzyme sequences, we only retained the embeddings of the first 1000 residues. Specifically, given an enzyme sequence of length L, represented by $\left(r_{1},r_{2},\text{…},r_{L}\right)$ where $r_{i}$ is the i‐th residue, ProtT5‐XL‐UniRef50 obtains the representation matrix $H\in R^{L\times d}$, where d=1,024 is the embedding dimension. The i‐th row of H corresponds to the embedding of the residue $r_{i}$.

#### 4.2.2 Reaction Representations

RXNFP model [36] takes the reaction SMILES format as input and converts catalytic reactions into vector representations, namely, reaction fingerprints, by leveraging Transformer architecture. Specifically, in addition to the individual SMILES tokens in a reaction, an extra token for the whole reaction is added. This token does not contribute any extra input information to the model. Instead, it serves to store information regarding the entire input reaction that is crucial for the downstream task. During training, all tokens, including the extra reaction token, are updated iteratively based on a self‐supervised learning strategy. The final embedding of the extra reaction token represents the reaction fingerprint, which captures intricate chemical relationships within the reaction. We denote the reaction fingerprint with $h_{r}\in R^{1\times k}$, where k=256 is the embedding dimension.

### 4.3 MCKcat Framework

#### 4.3.1 Extracting Multi‐Scale Convolutional Features

Because the dimensions of the pre‐trained representations of enzymes and reactions are different, we extracted hierarchical convolutional features from the pre‐trained representations of enzymes to align the enzyme embeddings and reaction representations, which were then used in the subsequent bidirectional cross‐attention block. Specifically, we adopted three parallel convolutional layers. The convolutional kernel sizes were (2, 1024), (3, 1024), and (4, 1024), respectively. The number of input channels was 1, and the number of output channels was set to num_filters = 256. We then concatenated these three convolutional outputs along the sequence dimension to obtain the feature matrix $M\in R^{(3L-6)\times 256}$. This design captured multi‐scale local feature information from enzyme sequences.

#### 4.3.2 Cross‐Attention Fusion Block

The appropriate integration of the enzyme residue embeddings and the reaction representations is crucial to our prediction. Our goal was to predict the $k_{\text{cat}}$ value of an enzyme‐catalyzed reaction, which can be interpreted as the influence of the active site of an enzyme on the corresponding catalytic reaction. Therefore, the integration module was responsible for cross‐talking between the reaction and the protein sequence context. Here, a cross‐attention fusion module was used to integrate these two representations. The concept of cross‐attention is derived from Perceiver, which is a variant of the Transformer architecture [37]. Perceiver performs excellently in handling multi‐modal inputs, being able to compress dimensions and improve computational speed. Previous studies have applied Perceiver to the prediction of compound‐protein interactions [38] as well as the prediction of functional phosphorylation sites and their regulatory types [39]. Here, we input the feature matrix M of the enzymes into the cross‐attention fusion block along with the reaction representation to obtain fully integrated embeddings for enzyme‐reaction combinations. Specifically, we set the query Q, the key K, and the value V with the reaction representation $h_{r}$, the enzyme feature matrix M, and the M, respectively, and obtained the updated reaction embeddings ${\hat{h}}_{r}$ as follows:

$$attention_{1}=softmax\left(\frac{h_{r}\cdot M^{T}}{\sqrt{C}}\right)$$

$${\hat{h}}_{r}=layer\_norm\left(attention_{1}\cdot M+h_{r}\right),$$

where C=256 is the embedding dimension and layer_norm is the layer normalization operation. Similarly, since the reaction is a fixed‐dimensional vector with sequence length 1, the attention weight from the enzyme to the reaction, $attention_{2}$, degenerates to an all‐one vector, i.e., $attention_{2}=1\in R^{(3L-6)\times 1}$. We obtained the updated enzyme feature matrix $\hat{M}$ with the following equation.

$$\hat{M}=layer\_norm\left(attention_{2}\cdot h_{r}+M\right).$$

Then, we performed the max pooling in $\hat{M}$ along the sequence dimension to obtain the vector representation of the enzyme, which was then concatenated with the updated reaction representation ${\hat{h}}_{r}$ to serve as the embedding for the enzyme‐reaction combination.

Finally, we input the embeddings of enzyme–reaction combinations into a three‐layer fully connected network. The numbers of neurons in the three layers are 256, 64, and 1, respectively, and the final output denotes the predicted $k_{\text{cat}}$ value. We adopted the mean squared error (MSE) loss function and the Adam optimizer for model training. The learning rate was set to $5\times 10^{-5}$, and the weight decay was set to $1\times 10^{-5}$.

To further enhance prediction performance and robustness, we adopted an ensemble learning strategy [13]. Specifically, we used 10 different random seeds to generate distinct initial model weights, trained 10 independent prediction models, and then averaged their outputs to obtain the final prediction results.

### 4.4 Baseline Methods

#### 4.4.1 TurNup

TurNup [12] first used a pre‐trained model ESM‐$1b_{ESP}$ [40] to obtain the enzyme representations. Here, ESM‐$1b_{ESP}$ was obtained by fine‐tuning the pre‐trained model ESM‐1b for the task of predicting the substrate scope of enzymes. Then, for the reaction information, the Differential Reaction Fingerprint (DRFP) [41] was used to obtain its representation. Specifically, DRFP first identifies the substructures of all substrates and products. Then, hash functions are used to map all substructures that exist only in the substrates or only in the products into a 2048‐dimensional binary fingerprint. Finally, the enzyme and reaction representations were fed into the gradient‐boosting model XGBoost [42], respectively, and the average of the prediction results was taken as the output of the final result.

#### 4.4.2 CatPred

The CatPred model [13] first used ESM‐2 (a pre‐trained protein language model) [43] and sequence attention to extract features of the enzyme sequence. The directed message passing neural network (D‐MPNN) [44] was adopted to extract the features of the substrate. Then the enzyme and reactant representations are concatenated together and input into a fully connected neural network to generate the mean and the variance. The negative log‐likelihood loss function was used in the CatPred model. Finally, CatPred used 10 different random number seeds to generate different initial weight parameters of the network, thereby creating 10 distinct prediction models. The final prediction result was obtained by taking the average of the output means of these prediction models.

#### 4.4.3 CataPro

CataPro encoded enzyme information using ProtT5, and substrate information using MolT5 [45] and RDKit [33]. For the substrate information encoding step, all reactants in the reaction were first concatenated and then filtered through a list of cofactors to select valid reactants. During the encoding process, the average value of all reactant embeddings was calculated and served as the final embedding of the substrate. Subsequently, the enzyme embedding and substrate embedding were concatenated, and a feed‐forward neural network was used to predict $k_{\text{cat}}$ values.

#### 4.4.4 EITLEM

A part of the research work on EITLEM [11] focused on the extraction of enzyme sequence features and substrate molecular features. The model used ESM2 (for enzyme sequence feature extraction) and RDKit (for substrate molecular feature extraction), respectively, to complete the feature extraction process. For the feature extraction of substrate molecules, all reactants in the reaction were first concatenated and then filtered through the list of cofactors, which served as the input for the RDKit model. Subsequently, the ProMolAtt and AttentionAgg algorithms were employed to perform adaptive fusion and transformation of the key features of enzymes and substrates, which constituted the core component of the $k_{\text{cat}}$ prediction module in their study.

### 4.5 Evaluation Metrics

To assess the performance of our model and other baseline methods, we adopted four evaluation metrics: *R* 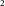, Pearson Correlation Coefficient (PCC), Spearman's rank correlation coefficient (SCC), and Mean Squared Error (MSE). Let $y_{i}$ denote the experimentally measured $k_{\text{cat}}$ value for data point i, ${\hat{y}}_{i}$ represent the predicted $k_{\text{cat}}$ value, $\bar{y_{t}}$ be the mean of the experimentally measured $k_{\text{cat}}$ values, $\bar{y}$ be the mean of the predicted $k_{\text{cat}}$ values, n denote the total number of samples, and $d_{i}$ be the rank difference between the predicted and experimental values for each data point.

$$R^{2}=1-\frac{\sum_{i=1}^{n}{\left(y_{i}-{\hat{y}}_{i}\right)}^{2}}{\sum_{i=1}^{n}{\left(y_{i}-\bar{y_{t}}\right)}^{2}}$$

$$\text{PCC}=\frac{\sum_{i=1}^{n}\left(y_{i}-{\bar{y}}_{t}\right)\left({\hat{y}}_{i}-\bar{y}\right)}{\sqrt{\sum_{i=1}^{n}{\left(y_{i}-{\bar{y}}_{t}\right)}^{2}}\sqrt{\sum_{i=1}^{n}{\left({\hat{y}}_{i}-\bar{y}\right)}^{2}}}$$

$$\text{SCC}=1-\frac{6\sum_{i=1}^{n}d_{i}^{2}}{n(n^{2}-1)}$$

$$\text{MSE}=\frac{1}{n}\sum_{i=1}^{n}{\left(y_{i}-{\hat{y}}_{i}\right)}^{2}$$

These metrics collectively provided a comprehensive evaluation of model performance, capturing both overall prediction accuracy and the strength of correlation between predicted and actual $k_{\text{cat}}$ values.

### 4.6 Computing Reaction Similarity

We first obtained the structural reaction fingerprints of the reactions. Specifically, first, a 1638‐dimensional binary molecular fingerprint (ExplicitBitVect) was calculated for each substrate or product. Then, a bitwise OR operation was performed on all the substrate fingerprints to generate a 1638‐dimensional binary vector that contains information about the substrates. Similarly, a bitwise OR operation was applied to all the product fingerprints to generate another 1638‐dimensional binary vector that contains information about the products. Finally, these two vectors were concatenated to obtain a 3276‐dimensional binary vector that provided the structural information of the reaction. Here, the function “Chem.rdChemReactions.CreateStructuralFingerprintForReaction” in RDKit was used to calculate these fingerprints. Next, for any two reactions, we used the Jaccard coefficient to measure the similarity between their corresponding 3276‐dimensional binary vectors and normalize it to the range of 0 to 1.

### 4.7 Experimental D*a*esign for Laccase Mutation

#### 4.7.1 Materials

Chemical 2,2
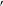
‐Azino‐bis(3‐ethylbenzothiazoline‐6‐sulfonic acid) (ABTS) was purchased from Sigma‐Aldrich (Shanghai, China). The Cycle‐Pure Kit, Gel Extraction Kit, and Plasmid Mini Kit I were sourced from Omega Bio‐tek (Norcross, GA, USA). Restriction enzymes, T4 DNA ligase, and DNA polymerase were obtained from TaKaRa Bio (Dalian, China). Site‐directed mutagenesis was performed using the KOD‐Plus‐Mutagenesis kit from Toyobo (Osaka, Japan). All other general chemicals and reagents were of analytical grade and obtained from commercial sources.

### 4.8 Plasmids, Strains, and Media

The pET‐28a(+) vector was used for gene expression. *Escherichia coli* JM109 served as the host for plasmid propagation and cloning, while *E. coli* BL21(DE3) was used for recombinant protein expression. All *E. coli* strains were cultivated at 37 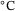 in Luria‐Bertani (LB) medium supplemented with 50 μg/mL kanamycin for plasmid maintenance.

### 4.9 Cloning of the Laccase Gene from *Bacillus aryabhattai* TCCC11368

The WT laccase gene (*balac*) was amplified from the genomic DNA of *Bacillus aryabhattai* TCCC11368 using primers summarized in Table S1. The sequence of the balAC was provided in Table S2. The PCR product and the pET‐28a(+) vector were digested with *Nco*I and *Xho*I, ligated, and transformed into *E. coli* JM109 for propagation. The resulting plasmid, pET‐*balac*, was confirmed by sequencing and subsequently transformed into *E. coli* BL21(DE3), yielding the expression strain *E. coli* BL21/pET‐*balac*.

### 4.10 Identification of the Residues for Laccase Mutation

AlphaFold2 (https://github.com/deepmind/alphafold) was utilized to model the structures of the WT baLAC [46, 47]. PoPMuSiC 2.1 (https://soft.dezyme.com/query/create/pop) was employed to calculate the solvent accessibility of each amino acid residue in the three‐dimensional structure of WT baLAC [48]. The MCKcat was used to determine the $k_{\text{cat}}$ values for potential baLAC mutants, using their amino acid sequences and the reaction SMILES representing laccase‐catalyzed oxidation (Table S3).

### 4.11 Site‐Directed Mutagenesis of Laccase

Site‐directed mutagenesis was performed via a one‐step PCR using primers summarized in Table S1. The pET‐*balac* plasmid harboring the WT baLAC gene was utilied as the template. After PCR amplification, the parental methylated plasmid DNA was digested with *Dpn*I, and the resulting product was transformed into *E. coli* DH5α. Positive clones carrying the intended mutations were verified by DNA sequencing. The confirmed mutant plasmids were then separately transformed into *E. coli* BL21(DE3) for recombinant protein expression.

### 4.12 Expression and Purification of Recombinant Laccase

A single colony of *E. coli* BL21(DE3) harboring either pET‐*balac* (for WT baLAC) or its mutant plasmids was inoculated into 5 mL of LB medium containing 50 μg/mL kanamycin and cultured at 37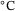 with shaking at 220 rpm for 12 h. Then, 1 mL of the seed culture was inoculated into 50 mL of fresh LB medium (with 50 μg/mL kanamycin) and grown under the same conditions until the ${\mathrm{OD}}_{600}$ reached 0.6. Protein expression was induced by 0.5 mM isopropyl‐β‐D‐1‐thiogalactopyranoside (IPTG), followed by incubation at 16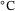 for 18 h. The recombinant WT baLAC and its mutants were purified as described previously [49], and protein concentration was determined using the BCA assay. Protein purity and molecular weight were confirmed by sodium dodecyl sulfate–polyacrylamide gel electrophoresis (SDS‐PAGE).

### 4.13 Measurement of Laccase Activity

The activity of WT baLAC and its mutants was assayed at 80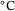 using ABTS as the substrate, following a previously reported method [50]. The reaction was performed in 0.1 M citrate–phosphate buffer (pH 5.0) with 6.25 mM ABTS, and the increase in absorbance at 420 nm ($\varepsilon_{420}$= 36 000 $M^{-1}{\mathrm{cm}}^{-1}$) was recorded. One unit of laccase activity was defined as the quantity of enzyme required to oxidize 1 μmol of ABTS per minute.

### 4.14 Kinetics Analysis of Laccase

The kinetic parameters of WT baLAC and its mutants were determined using ABTS at concentrations ranging from 1.25 to 12.5 mM. The $k_{\text{cat}}$ values were derived from nonlinear regression analysis of the Michaelis–Menten curves using Origin 9.0 (OriginLab, Northampton, MA, USA).

### 4.15 Determination of Thermostability of Laccase

The thermostability of WT baLAC and its mutants was assessed by incubating the enzymes at 80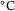 in 0.1 M citrate–phosphate buffer (pH 5.0). Aliquots of the samples were collected at specific time intervals, cooled, and assayed for residual activity following the standard procedure noted in the “Measurement of laccase activity” section. The half‐life ($t_{1/2}$) was determined by fitting the natural logarithm of residual activity versus incubation time to a linear regression model.

## Author Contributions

L.W. and Y.L. conceived of the study, designed the experiments, and edited the manuscript. F.G. performed the computational experiments and wrote the manuscript. X.M. performed the wet‐lab experiments and wrote the manuscript. J.L., M.W. and F.L. participated in the data analysis. All authors read and approved the final manuscript.

## Funding

This work was supported by the National Natural Science Fund of China (32530082), the Key Research and Development Program of Ningxia Hui Autonomous Region of China (2024BEE02021), and the Tianjin Outstanding Youth Science Fund of China (22JCJQJC00030).

## Conflicts of Interest

The authors declare no conflicts of interest.

## Supporting information


**Supporting File**: advs77789‐sup‐0001‐SuppMat.docx.

## Data Availability

The TurNup‐DB dataset is derived from TurNup [10.1038/s41467‐023‐39840‐4], which integrates enzyme‐reaction‐turnover number ($k_{\text{cat}}$) combination data from the Sabio‐RK [http://sabio.h‐its.org/], UniProt [https://www.uniprot.org/], and BRENDA [https://www.brenda‐enzymes.org/]. The MCKcat‐DB dataset constructed in this study is built by integrating datasets from CatPred [10.1038/s41467‐025‐57215‐9] and CataPro [10.1038/s41467‐025‐58038‐4]. The Deep Mutational Scanning (DMS) datasets used to validate the enzyme mutant ranking ability of MCKcat are sourced from public studies: the TEM‐1 (β‐lactamase) DMS dataset is derived from 10.1093/molbev/msu081, the BGL3 (β‐glucosidase) DMS dataset from 10.1073/pnas.1422285112, and the HIS3 DMS dataset from 10.1371/journal.pgen.1008079. The MCKcat‐DB, DMS datasets, and TurNup‐DB have been publicly deposited in the Zenodo repository at [https://zenodo.org/records/21852206] and the GitHub repository at [https://github.com/gefengya/MCKcat]. AlphaFold2 and PoPMuSiC 2.1 are available from https://github.com/deepmind/alphafold and https://soft.dezyme.com/query/create/pop, respectively. The web server for MCKcat is available at http://117.72.109.140:5000/about.
