# Supplementary material for: Predicting Enzyme Turnover Numbers and Enabling Rational Enzyme Evolution
Source: Adv Sci (Weinh). 2026 Sep 27:e77789. Online ahead of print. doi: 10.1002/advs.77789 (PMC13616300; doi:10.1002/advs.77789)
Supplement: Supplementary file 1 [file ADVS-9999-e77789-s001.docx]

**Supplementary Materials**

1. Ablation experiments

Figure S1. Quantitative ablation analysis of multi-scale convolution and cross-attention modules on TurNup-DB using R^2^. **A** Comparison results under the unseen enzyme prediction scenario. **B** Performance metrics obtained under the unseen reaction prediction scenario. Each colored bar corresponds to one ablation configuration, with black error bars indicating the standard deviation across five independent data splits.

2. The 3D structure of balAC

Figure S2. The Positional distribution of the 34 candidate mutation sites in the 3D structure of baLAC.

3. Soluble expression analysis of baLAC mutants

Figure S3. Validation of soluble expression for baLAC mutants with the highest predicted *k*_cat_ values. **M**: Mark, **1**: The lysate supernatant of the pET-28a(+) empty vector strain, **2**: The lysate supernatant of the baLAC mutants (**A**: Y495D, **B**: Y495E, **C**: S497P, **D**: Y495P, **E**: Y495R, **F**: N493P, **G**: N498P, **H**: Y495K, **I**: Y495H, **J**: R492P, **K**: K494P, **L**: N356F, **M**: K496H, **N**: K359D, **O**: N498D, **P**: S497D, **Q**: K496D, **R**: K359E, **S**: K359F, and **T**: N498E).

Figure S4. Validation of soluble expression for baLAC mutants with the lowest predicted *k*_cat_ values. **M**: Mark, **1**: The lysate supernatant of the pET-28a(+) empty vector strain, **2**: The lysate supernatant of the baLAC mutants (**A**: N493R, **B**: N493K, **C**: Q428P, **D**: F334E, **E**: P338E, **F**: Q428F, **G**: Y218E, **H**: P408H, **I**: P408E, and **J**: N429P).

4. Purification of baLAC mutants

Figure S5. SDS-PAGE analysis of purified baLAC mutants with the highest predicted *k*_cat_ values. **M**: Mark, **1**: The purified baLAC mutants (**A**: Y495D, **B**: Y495E, **C**: S497P, **D**: Y495P, **E**: Y495R, **F**: N493P, **G**: N498P, **H**: Y495K, **I**: Y495H, **J**: R492P, **K**: K494P, **L**: N356F, **M**: K496H, **N**: K359D, **O**: N498D, **P**: S497D, **Q**: K496D, **R**: K359E, **S**: K359F, and **T**: N498E).

Figure S6. SDS-PAGE analysis of purified baLAC mutants with the lowest predicted *k*_cat_ values. **M**: Mark, **1**: The purified baLAC mutants (**A**: N493R, **B**: N493K, **C**: Q428P, **D**: F334E, **E**: P338E, **F**: Q428F, **G**: Y218E, **H**: P408H, **I**: P408E, and **J**: N429P).

Figure S7. Prediction and virtual screening of hotspot residues for targeted mutagenesis to reduce *k*_cat_ values. **A** Predicted log10*k*_cat_ values for the bottom 10 hits by using MCKcat. **B** Relative *k*_cat_ values and half-life of the WT baLAC and its mutants.

5. Interactive interface for prediction using the MCKcat model

Figure S8. User interface for deploying the MCKcat model. The interface requires input of an enzyme's amino acid sequence, along with characterization data for all substrates and products. Substrate/product characterization can be provided using either SMILES strings or InChI strings. For batch processing capabilities, users need upload a CSV file containing multiple reactions to execute calculations in bulk.

6. Primer sequences used in the experiments

Table S1. The primers used in this study.

| Name | Sequence（5’-3’） |
| --- | --- |
| F | TTAAGAAGGAGATATACCATGGGC |
| R | TGGTGGTGGTGGTGCTC |
| Y495D-R1 | GTTTGATTTGTCTTTATTACGATT |
| Y495D-R2 | TGGTGGTGGTGGTGCTCGAGGTTTGATTTGTCTTTATTACGATT |
| Y495E-R1 | GTTTGATTTCTCTTTATTACGATT |
| Y495E-R2 | TGGTGGTGGTGGTGCTCGAGGTTTGATTTCTCTTTATTACGATT |
| S497P-R1 | GTTGGGTTTGTATTTATTACGATTGG |
| S497P-R2 | TGGTGGTGGTGGTGCTCGAGGTTGGGTTTGTATTTATTACGATTGG |
| Y495P-R1 | GTTTGATTTGGGTTTATTACGATT |
| Y495P-R2 | TGGTGGTGGTGGTGCTCGAGGTTTGATTTGGGTTTATTACGATT |
| Y495R-R1 | GTTTGATTTGCGTTTATTACGATT |
| Y495R-R2 | TGGTGGTGGTGGTGCTCGAGGTTTGATTTGCGTTTATTACGATT |
| N493P-R1 | GTTTGATTTTCATTTTCTACGATT |
| N493P-R2 | TGGTGGTGGTGGTGCTCGAGGTTTGATTTTCATTTTCTACGATT |
| N498P-R1 | GGGTGATTTTCATTTATTACGATT |
| N498P-R2 | TGGTGGTGGTGGTGCTCGAGGGGTGATTTTCATTTATTACGATT |
| Y495K-R1 | GTTTGATTTCTTTTTATTACGATT |
| Y495K-R2 | TGGTGGTGGTGGTGCTCGAGGTTTGATTTCTTTTTATTACGATT |
| Y495H-R1 | GTTTGATTTGTGTTTATTACGATTGG |
| Y495H-R2 | TGGTGGTGGTGGTGCTCGAGGTTTGATTTGTGTTTATTACGATTGG |
| N492P-R1 | GTTTGATTTTACTTTATTGGGATT |
| N492P-R2 | TGGTGGTGGTGGTGCTCGAGGTTTGATTTTACTTTATTGGGATT |
| K494P-R1 | GTTTGATTTTACGGGATTACGATT |
| K494P-R2 | TGGTGGTGGTGGTGCTCGAGGTTTGATTTTACGGGATTACGATT |
| N356F-F | CGTATTCCATTATTTAAAGTGAAAAAGA |
| N356F-R | TCTTTTTCACTTTAAATAATGGAATACG |
| K496H-R1 | GTTTGAGTGGTATTTATTACGATTGG |
| K496H-R2 | TGGTGGTGGTGGTGCTCGAGGTTTGAGTGGTATTTATTACGATTGG |
| K359D-F | GTGGACAAGATAAGACGATTGAC |
| K359D-R | GTCAATCGTCTTATCTTGTCCAC |
| N498D-R1 | GTCTGATTTTACTTTATTACGATT |
| N498D-R2 | TGGTGGTGGTGGTGCTCGAGGTCTGATTTTACTTTATTACGATT |
| S497D-R1 | GTTGTCTTTTACTTTATTACGATT |
| S497D-R2 | TGGTGGTGGTGGTGCTCGAGGTTGTCTTTTACTTTATTACGATT |
| K496D-R1 | GTTTGAGACGTATTTATTACGATTGG |
| K496D-R2 | TGGTGGTGGTGGTGCTCGAGGTTTGAGACGTATTTATTACGATTGG |
| K359E-F | TTAAATAAAGTGGAAAAGATAAGACGATT |
| K359E-R | AATCGTCTTATCTTTTCCACTTTATTTA |
| K359F-F | TTAAATAAAGTGTTCAAGATAAGACGATT |
| K359F-R | AATCGTCTTATCTTGAACACTTTATTTAA |
| N498E-R1 | CTCTGATTTTACTTTATTACGATT |
| N498E-R2 | TGGTGGTGGTGGTGCTCGAGCTCTGATTTTACTTTATTACGATT |
| F334E-F | CCAAGTAACTAAAGAGTTATCTTGTCCTG |
| F334E-R | CAGGACAAGATAACTCTTTAGTTACTTGG |
| Y218E-F | CTAATTTACCAGAGCCTTCAATTGT |
| Y218E-R | ACAATTGAAGGCTCTGGTAAATTAG |
| P408H-F | GAATTTAACCCACGGAATTCAT |
| P408H-R | ATGAATTCCGTGGGTTAAATTC |
| N493R-R1 | GTTTGATTTGTATTTACTACGATTGGAAG |
| N493R-R2 | TGGTGGTGGTGGTGCTCGAGGTTTGATTTGTATTTACTACGATTGGAAG |
| N493K-R1 | GTTTGATTTGTATTTACTACGATTGGAAG |
| N493K-R2 | TGGTGGTGGTGGTGCTCGAGTGATTTGTATTTAAGACGATTGGAAG |
| Q428P-F | TCGTTATGATCCAAATGGAAACT |
| P338E-F | TTTATCTTGTGAAGATCAGAGCAGA |
| P338E-R | TCTGCTCTGATCTTCACAAGATAAA |
| P408E-F | TGAATTTAACCGAAGGAATTCATC |
| P408E-R | GATGAATTCC TTCGGTTAAA TTCA |
| Q428F-F | TCGTTATGATTTCAATGGAAACTT |
| Q428F-R | AAGTTTCCATTGAAATCATAACGA |
| N429P-F | TATGATCAACCTGGAAACTTGGT |
| N429P-R | ACCAAGTTTCCAGGTTGATCATA |

7. Sequence of WT baLAC

Table S2. Wild-type baLAC sequence.

MELKKFVDILPIPSIIRPKGMYKGRPFYDVCMIETLHKFHRDLPKTKVWGYNGLVPGPTFNVEKNQPIYVRWANNLPEKHFLPVDKTIHGASHNPEVRTVVHLHGSPSEPASDGHPEAWFTRGFRQTGPHFMKEIYHYTNGERATALWYHDHALGVTRLNVYAGLAGLYFIRDEQERSLSLPKGKYEVPLIIQDKTFNPDGSLFYPAQPDNPSPNLPYPSIVPSFLGDTITVNGKVWPFLKVEPRKYRFRLLNASNTRTYQFQLSNLRPFALIGTDGGLLSRPIKVKSLDVSPAERIDIVIDFSGLEGKNVILQDGFESENPTGEIMEFQVTKFLSCPDQSRLPPLLSHIDRIPLNKVKKIRRLTLNDSQDEFGRLMLLFDDKEWMNPATETPLLNSVEIWELVNLTPGIHPIHVHLVNFRVLDRYDQNGNLVAPLPADFGLKDTVLVGPGETVRIIMKFQPFSGDYVWHCHRLEHEDHDMMRPLKIIPSNRNKYKSN

8. Reaction smiles for laccase-catalyzed oxidation reaction

Table S3. SMILES of substrates/products in laccase-catalyzed oxidation.

2CCN1C2=C(C=C(C=C2)S(=O)(=O)O)SC1=NN=C3N(C4=C(S3)C=C(C=C4)S(=O)(=O)O)CC.O=O>>2CCN\1C2=C(C=C(C=C2)S(=O)(=O)[O-])S/C1=N\N=C\3/[N+](C4=C(S3)C=C(C=C4)S(=O)(=O)[O-])CC.2O
